# Experimental Verification of Dielectric Models with a Capacitive Wheatstone Bridge Biosensor for Living Cells: *E. coli*

**DOI:** 10.3390/s22072441

**Published:** 2022-03-22

**Authors:** Faezeh Zarrinkhat, Luís Jofre-Roca, Marc Jofre, Juan M. Rius, Jordi Romeu

**Affiliations:** 1 CommSensLab, Universitat Politecnica de Catalunya (UPC), 08034 Barcelona, Spain; luis.jofre@upc.edu (L.J.-R.); marc.jofre@upc.edu (M.J.); juan-manuel.rius@upc.edu (J.M.R.); jordi.romeu-robert@upc.edu (J.R.); 2 Department of Research and Innovation, Fundació Privada Hospital Asil de Granollers, 08402 Granollers, Spain

**Keywords:** dielectric spectroscopy, *E. coli* bacteria, Maxwell–Garnet model, Maxwell–Wagner theory

## Abstract

Detection of bioparticles is of great importance in electrophoresis, identification of biomass sources, food and water safety, and other areas. It requires a proper model to describe bioparticles’ electromagnetic characteristics. A numerical study of *Escherichia coli* bacteria during their functional activity was carried out by using two different geometrical models for the cells that considered the bacteria as layered ellipsoids and layered spheres. It was concluded that during cell duplication, the change in the dielectric permittivity of the cell is high enough to be measured at radio frequencies of the order of 50 kHz. An experimental setup based on the capacitive Wheatstone bridge was designed to measure relative changes in permittivity during cell division. In this way, the theoretical model was validated by measuring the dielectric permittivity changes in a cell culture of *Escherichia coli* ATTC 8739 from WDCM 00012 Vitroids. The spheroidal model was confirmed to be more accurate.

## 1. Introduction

Bacteria detection and cell analysis play an important role in regular microbiology procedures [1], and they also have significant applications for bioenergy in order to identify new sources of biomass and increase efficiency in many biotechnology processes [2]. Bacteria-related diseases that are caused by contaminated food or water threaten public health, and their prevention requires reliable, convenient, and efficient sensing methods [3]. Sensing techniques based on the electrical properties of bioparticles have attracted much attention. Despite being electrically neutral, all living organisms include charged ions, polarized molecules, and electrical fields. In recent years, the exploitation of their intrinsic electrical properties has emerged as an appealing approach for concentrating and detecting bacteria [1]. Various electrical sensing technologies have been developed, including micro-fabrication technology, nano-fabrication technology [4], and microwave microorganism detection technology, based on microfluidic platforms that are used to sense the membrane potential of bacteria [5,6]. Furthermore, bacteria trapping and detection can be implemented with lab-on-chip devices [7].

Many studies have shown the roles and effects of static and pulsed radio frequencies and millimeter and terahertz waves on various biomolecules, cells, and tissues [8]. It is possible to monitor the functionality of an organism according to its electromagnetic properties. The most accessible example is the resting potential in neurons; from its absence, a lack of active functionality or even the death of a neuron can be concluded [9]. Cancer cells demonstrate lower resting potential in comparison to healthy ones [10]. The design and assessment of electrical-based bio-sensors require an analytic model to define the electrical properties of bioparticles. On the other hand, electrophoresis, which has broad applications, including clinical, biological, single-cell analysis, environmental, pharmaceutical, and food analysis applications [11], requires an accurate dielectric cell model [12]. Therefore, there is a need to have accurate models for the dielectric properties of cells. An extensive review of the models for the dielectric properties of cells can be found in [13,14]. In this paper, the Maxwell–Wanger theory is applied; it leads to the accurate modeling of the dielectric permittivity—referred to in this paper as permittivity—of different layers of spheroidal bacteria [15], and it is used to model the dielectric properties of the interface between cell membranes and the outer medium [16]. In the particular case of spherical particles, the Maxwell–Garnett theory leads to a simpler formulation [17].

Here, among different microorganisms, *E. coli* bacteria became candidates for the analysis due to their straightforward geometry and the simplicity of experimenting with them. In Section 2, the use of the Maxwell–Wanger and Maxwell–Garnett theories to model them as multilayer spheroidal and spherical elements is presented. In Section 4, the experimental setup, the calibration procedure, and the experimental results of the changes in the effective permittivity during cell culture growth are presented. The objective is to assess the accuracy of the models in predicting the dielectric properties of the cells.

## 2. Theory

**Figure 1:**
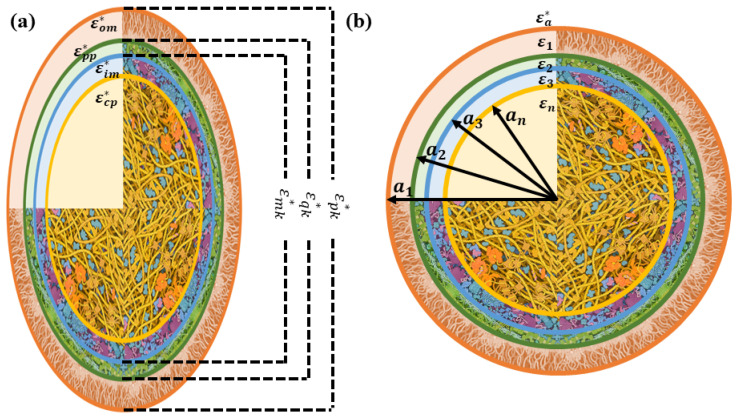
Structure of (**a**) a coated prolate spheroid and (**b**) a coated sphere resembling *E. coli* in a culture. The cell image was derived from an illustration by David S. Goodsell, RCSB Protein Data Bank.

In this section, two different geometrical models for the *E. coli* cells are considered. In particular, a coated sphere and a coated spheroid, as shown in Figure 1, were used to derive a model for the dielectric permittivity of an *E. coli* culture.

### 2.1. Modeling *E. coli* Bacterial Culture with Prolate Spheroidal Particles

The basic structure of *E. coli* includes four parts: the cytoplasm, inner membrane, periplasm, and outer membrane. The Maxwell–Wagner interfacial polarization theory allows one to obtain the relative permittivity of a culture of *E. coli* bacteria in a medium by solving the Laplace equation in the ellipsoidal coordinates for a three-shelled ellipsoid, as shown in Figure 1a. An equation to define the effective permittivity of a culture consisting of prolate spheroidal particles randomly dispersed in a continuous medium $\varepsilon_{cul}^{*}$ is [15]:

(1) $$\varepsilon_{cul}^{*}=\varepsilon_{a}^{*}\frac{2\rho \sum_{k=x,y,z}\frac{\varepsilon_{pk}^{*}-\varepsilon_{a}^{*}}{\alpha_{k}\varepsilon_{pk}^{*}+\left(1-\alpha_{k}\right)\varepsilon_{a}^{*}}+9}{9-\rho \sum_{k=x,y,z}\frac{\varepsilon_{pk}^{*}-\varepsilon_{a}^{*}}{\alpha_{k}\varepsilon_{pk}^{*}+\left(1-\alpha_{k}\right)\varepsilon_{a}^{*}}}$$

where ρ and $\varepsilon_{pk}^{*}$ are the volume fraction of *E. coli* and the equivalent complex relative permittivity of the shell-covered ellipsoid along the *k* axis, where k=x,y,z. In addition, $\varepsilon_{a}^{*}$ and $\alpha_{k}$ are the complex relative permittivity of the external medium, and α represents the depolarization factors along the *k* axis. The relative complex permittivity is $\varepsilon^{*}=\varepsilon -\frac{j\sigma }{\omega \varepsilon_{0}}$, where ε is referred to as the relative permittivity and σ as the conductivity. The details of the theory and variables are thoroughly addressed in [13,15,18].

### 2.2. Modeling *E. coli* Bacterial Culture with Spherical Particles

To model the effective permittivity of an *E. coli* bacterial culture as a spherical particle, the Maxwell–Garnett theory, which is a classical mixing approach [17,19], is applied. For a culture consisting of multi-layered inclusions, the effective permittivity can be found as follows:

(2) $$\varepsilon_{cul}^{*}=\varepsilon_{a}^{*}+\frac{\frac{\alpha_{cul}}{V_{1}}}{1-\frac{\alpha_{cul}}{3\varepsilon_{a}^{*}V_{1}}}$$

where $V_{1}$ is the volume of the external sphere and $\alpha_{cul}$ is the culture’s polarizability, which is defined as a function of $\varepsilon_{i}$ and $a_{i}$, the permittivity and radius of the *i*th (*i* = 1:*n*) layer in each collusion, as shown in Figure 1b.

## 3. Mimicking the Functional Activity of Microorganisms: Duplication Process

**Figure 2:**
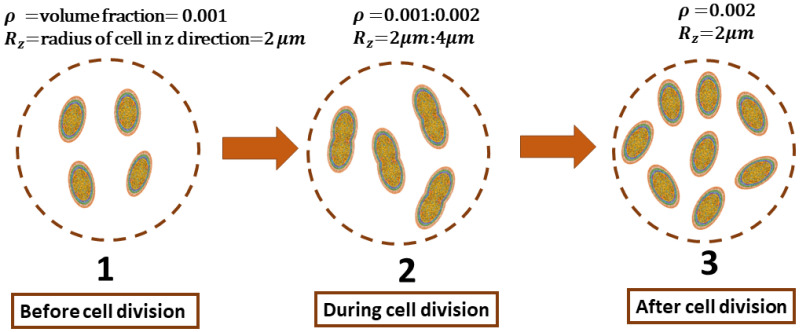
The duplication procedure in three phases: before cell division, during cell division, and after cell division.

The functional activity of growth in *E. coli* is considered as the procedure of duplication of the cells in the culture. The meiosis (cell division) process includes three stages, as shown in Figure 2. In stage one, before cell division, all cells in the culture have a volume fraction ρ, and the radius of the ellipsoidal cell in the *z*-direction is $R_{z}$. As elaborated in [20], only the length of the cells experiences changes while duplicating. The second stage is defined by the moment just before duplication happens in which ρ and $R_{z}$ are doubled. Finally, in the last stage, duplication has been finished. The culture’s volume fraction is 2ρ, and $R_{z}$ is the same as in the beginning.

Electromagnetic changes in the *E. coli* culture are investigated from stage 1 to 2 and from stage 1 to 3 through the formulation introduced in Section 2. The frequency range from 0.1 to 1000 kHz is considered. The initial values are $\rho_{0}=0.001$ and $R_{z0}=2$ μm. In the following simulations, the complex permittivity and dimension of each layer of *E. coli* bacteria are set according to [21].

**Figure 3:**
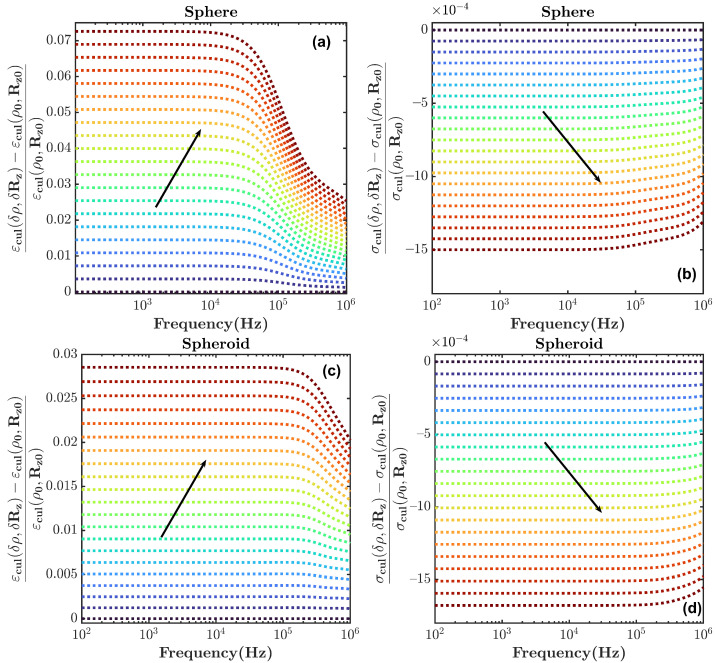
Normalized changes in permittivity and conductivity of an *E. coli* culture computed with the (**a**,**b**) spherical model and (**c**,**d**) spheroidal model. In the transition from phase 1 to phase 2, ρ and $R_{z}$ are doubled with 21 uniform steps. The different lines correspond to the variation of the total permittivity or conductivity after each step. The black arrow indicates the direction of the progression of time. The transition starts from the blue-shaded area and ends in the red-shaded area.

To begin with, Figure 3a,b are computed with Equation (2), showing changes in the permittivity and conductivity of the culture when it experiences the transition from phase 1 to phase 2; the volume fraction ρ and radius of the cells in the *z* direction $R_{z}$ increase by two times in 21 uniform steps. In these plots, *E. coli* bacteria are modeled as a three-layered sphere. Additionally, Figure 3c,d show the same variation from phase 1 to 2 when considering *E. coli* bacteria as a three-layered spheroid in the culture according to Equation (1). Notice that the relative changes with respect to the culture medium are displayed. Figure 3 shows the variation in the dielectric properties of the culture medium when $R_{z}$ and ρ are doubled. The process is subdivided into 21 regular time intervals. Each curve of Figure 3 corresponds to the relative dielectric change at each one of the time intervals. The arrow shows the time progression. The complex culture permittivity $\varepsilon_{cul}^{*}\left(\delta \rho ,\delta R_{z}\right)$ is obtained for each time interval. The culture permittivity $\varepsilon_{cul}$ and conductivity $\sigma_{cul}$ are computed from $\varepsilon_{cul}^{*}=\varepsilon_{cul}+\frac{i\sigma_{cul}}{2\pi f\varepsilon_{0}}$, where *f* is the frequency and $\varepsilon_{0}$ is the vacuum permittivity. Finally, the changes in permittivity and conductivity are normalized by the permittivity and conductivity of the culture at the beginning of the transition ($\varepsilon_{cul}\left(\rho_{0},R_{z0}\right)$ and $\sigma (\rho_{0},R_{z0})$, respectively). It is shown that changes in the real part of the permittivity are more significant than those in the conductivity, and the spherical model predicts a higher change in the real part of the permittivity than in the spheroidal one.

**Figure 4:**
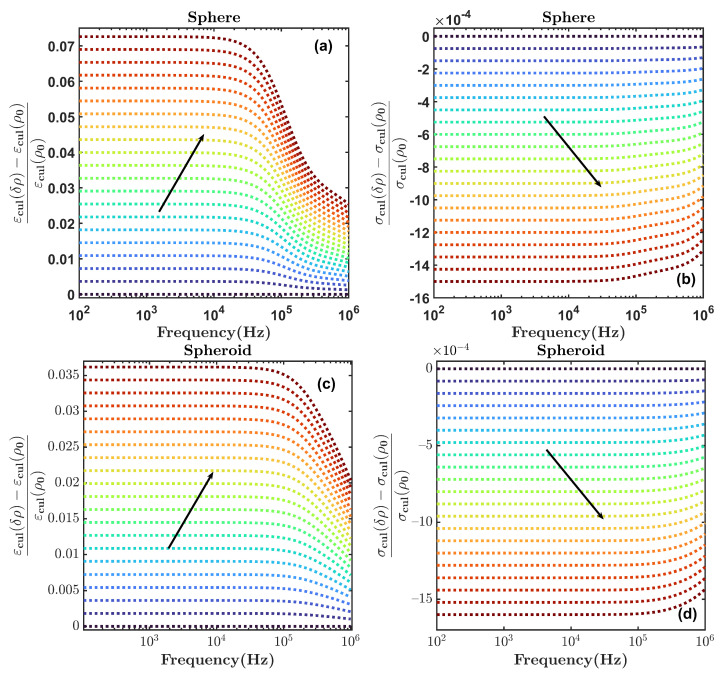
Normalized changes in permittivity and conductivity of an *E. coli* culture computed with the (**a**,**b**) spherical model and (**c**,**d**) spheroidal model. In the transition from phase 1 to phase 3, ρ is doubled with 21 uniform steps, and $R_{z}$ remains constant. The black arrow indicates the direction of the progression of time. The transition starts from the blue-shaded area and ends in the red-shaded area.

The modeling of *E. coli* with the spherical and spheroidal models for the transition from phase 1 to phase 3 is shown in Figure 4, with a similar procedure to that of Figure 3, although only ρ is doubled at each frequency point. As is shown, the variation of the permittivity is greater than that of the conductivity in both models. In addition, the changes for the transition from phase 1 to 3 are more significant than those for the transitions from phase 1 to 2. The analysis also shows that the greatest changes occur at frequencies below 100 kHz, and they are practically frequency-invariant below that frequency.

## 4. Experimental System and Procedures

### 4.1. System Description

**Figure 5:**
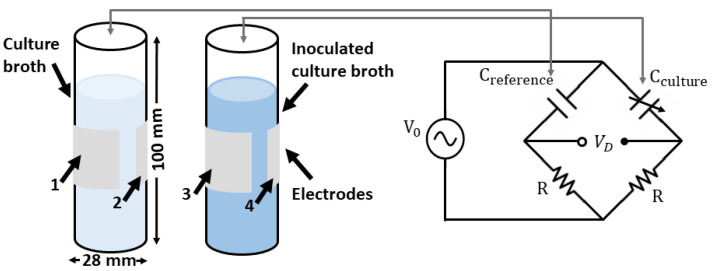
Principle of the measurement setup based on a Wheatstone bridge; the culture broth in the flask covered with electrodes serves as a reference capacitance, and the flask filled with inoculated *E. coli* is a variable capacitance. The diameter of each flask is 28 mm, and the height is 100 mm. The four electrodes are labeled 1, 2, 3, and 4.

In order to measure the dielectric properties of *E. coli* cells, an experimental setup was implemented to monitor the macroscopic changes in the dielectric constant of a cell culture’s growth. In [22], the optical density at 600 nm (OD 600) was used to monitor the cell culture growth in a dynamic way. The transparent culture media transformed into saturated opaque media as the cell concentration increased. The cell concentration was measured via the optical absorption at 600 nm with periodic measurements during the 8–12 h period of cell growth with a sensitivity that started at a cell concentration of about $10^{7}$ cells/mL. These experimental data were used as a reference to define some of our system parameters, such as the measurement time, cell growth rate, and sensitivity target. Our proposed measurement setup was based on the well-known capacitive Wheatstone bridge. As shown in Figure 5, a capacitor was formed by placing electrodes around a culture flask consisting of a 50 mL polypropylene test tube. A reference flask was filled with the culture medium, while the second flask was also filled with a culture medium that was inoculated with *E. coli*. As the cell culture grew, the dielectric constant of the medium changed, producing a variation in the capacitance.

**Figure 6:**
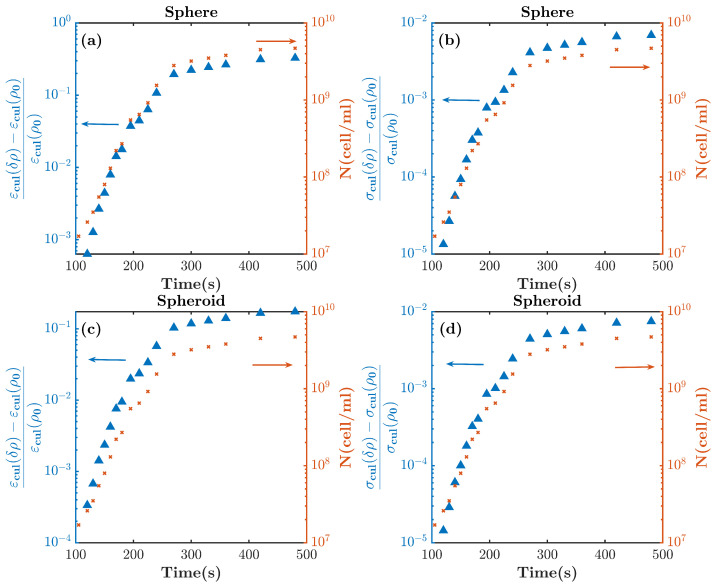
Expected relative changes in the dielectric properties of the growth of the cell culture (blue triangles are related to the left *y*-axis) and the cell concentration (the red x is related to the right *y*-axis) over time. The relative variations in (**a**) the real part of the permittivity and (**b**) the conductivity of the spherical model, as well as the relative variations in (**c**) the real part of the permittivity and (**d**) the conductivity of spheroidal model, are shown.

Given the results in Figure 3 and Figure 4, and assuming a similar growth rate to the one reported in [22], Figure 6 shows the expected relative changes in dielectric permittivity and conductivity for the inoculated cell culture broth over time at a frequency of 50 kHz. It was observed that the relative changes in conductivity were about one order of magnitude smaller than the changes in permittivity. On the other hand, it was shown that, depending on the cell model, the relative changes in dielectric permittivity of between $10^{-4}$, and $10^{-3}$ with respect to the culture medium corresponded to a cell concentration of $10^{7}$ cells/mL. In order to relate the volume fraction ρ with the cell concentration, we considered a cell-occupied volume of $V_{cells}\approx N\mu$m${}^{3}\times$ (1 mL/10${}^{12}\mu$m${}^{3})=N$× 10${}^{-12}$ (mL), and *N* (cell/mL) was the cell concentration.

In order to properly design the capacitive bridge electronics, it was necessary to have an estimate of the capacitance of the flasks. A simulation was conducted with the CST Low-Frequency Quasistatic solver to evaluate the capacitance of the water-filled flasks in the arrangement shown in Figure 5. The two flasks were filled with distilled water with a permittivity of 78.4 and tanδ 0.025 at 50 kHz. The flasks were positioned over a grounded conductor and were modeled as cylinders with a radius of 14 mm, a height of 100 mm, a thickness of 1 mm, and a dielectric permittivity of 2.1. The resulting capacitance matrix showed a capacitance of 34 pF between electrodes 1, 2, 3, and 4; the capacitance between electrodes and the ground plane was of the order of 5 pF, and the capacitance between crossed electrodes was of the order of 1 pF.

An additional look at where electric energy was stored showed that similar amounts of energy were stored in the liquid inside the flask and in the flask itself, and the conductivity between electrodes 1 and 2 was $2.57\times 10^{-7}$ S. Finally, the capacitance was measured with an Agilent 4263B impedance meter. The measured capacitance was 28 pF for the flask filled with distilled water at 20 ${}^{\circ }$C and 16 pF for the empty flask. These results are in accordance with the simulated ones, and differences can be attributed to the series capacitive effect of the adhesive electrodes, which slightly reduced the measured capacitance, and the uncertainty of the exact value of the dielectric constant of the plastic material employed to build the flask. Overall, the liquid-filled flask could be modeled as a capacitor in the form:

(3) $$C_{flask}=C_{st}+C_{0}\left(1+x\right)=C_{st}+C_{0}\left(1+\frac{\epsilon_{r}^{\prime }\left(y\right)}{\epsilon_{r}}y\right)$$

where *x* is the relative change in the dielectric constant of the liquid, $C_{st}$ is the capacitance associated with the fields in the surroundings, and $C_{0}$ is the nominal flask capacitance, which was assumed to be invariant with respect to changes in the dielectric constant of the liquid; the prime symbol stands for the derivative. In the case that the dielectric permittivity of the medium inside the flask changes according to the external variable *y*, small variations can be linearized, as shown in Equation (3). The two flasks were placed in the bridge (Figure 5), and it was assumed that one had a constant capacitance and the other had a variable capacitance due to the changes in the dielectric constant of the liquid caused by the cell growth. This configuration is known as a quarter-bridge, as only one of the impedances of the bridge is sensitive to external variables. The sensitivity of the bridge is maximized when the impedance of the resistor and the capacitor in the bridge branch are the same [23]. Considering the values of the measured flask capacitance and an operating frequency of 50 kHz, a resistor of 150 KΩ was used in series with a 10 kΩ adjustable resistor in the bridge. For small changes, the differential voltage was derived as follows [24]:

(4) $$\frac{V_{d}}{V_{0}}\approx \frac{x}{4}=\frac{1}{4}\frac{\epsilon_{r}^{\prime }\left(y\right)}{\epsilon_{r}}y$$

A differential amplifier based on the INA 114 chipset by Texas Instruments was employed. The limiting factor in the bridge sensitivity was the common-mode rejection ratio (CMRR), which is defined as the ratio between the amplifier’s differential gain and the common-mode gain. For the INA 114, the CMRR at 50 kHz was 83 dB for a voltage gain of 10. According to the measurement setup in Figure 5, the common-mode voltage is $V_{0}/2$, and assuming a differential voltage, as in Equation (4), the measured voltage is

(5) $$V_{out}=\frac{V_{0}}{2}GCM+\frac{V_{0}}{4}xGD$$

where GCM and GD are the common mode and differential gain, respectively. In order to measure the changes in the dielectric constant, let us assume that

(6) $$\frac{xGD}{4}>10\frac{GCM}{2}$$

which assumes that the desired signal is 20 dB above the unwanted signal. A condition that leads to a CMRR with limited sensitivity is given by:

(7) $$x_{min}\approx 10\frac{2}{CMRR}$$

**Figure 7:**
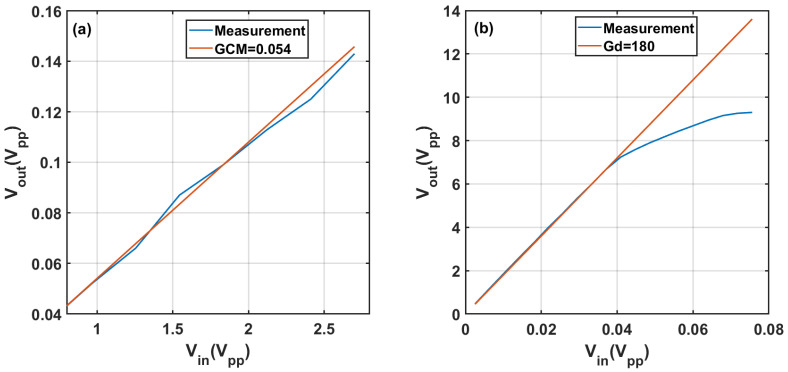
Measured (**a**) common-mode and (**b**) differential-mode gain of the amplifier at 50 kHz.

The differential and common-mode gain of the amplifier were measured, and the results are shown in Figure 7a,b, respectively. In the experimental setup, the INA 114 differential amplifier was followed by a TL071 amplifier in order to increase the signal level. At the frequency of interest of 50 kHz, the overall differential gain was 180 and the common-mode gain was 0.054, with a resulting CMRR of 70 dB; the resulting sensitivity was of the order of $x_{min}\approx 6\times 10^{-3}$, which was of the order of magnitude of the expected changes in the dielectric constant for a cell concentration of $10^{7}$ cells/mL, as shown in Figure 6. In order to perform the measurement, a Rhode and Schwartz RTC1000 Oscilloscope was used to acquire the signal, and its internal generator was used as a source signal. The capacitance measurement approach was prone to picking up electromagnetic noise from surrounding equipment, especially lighting systems. A noise-level evaluation of the system is addressed in Appendix A.1.

### 4.2. Cell Culture Growth Measurements

**Figure 8:**
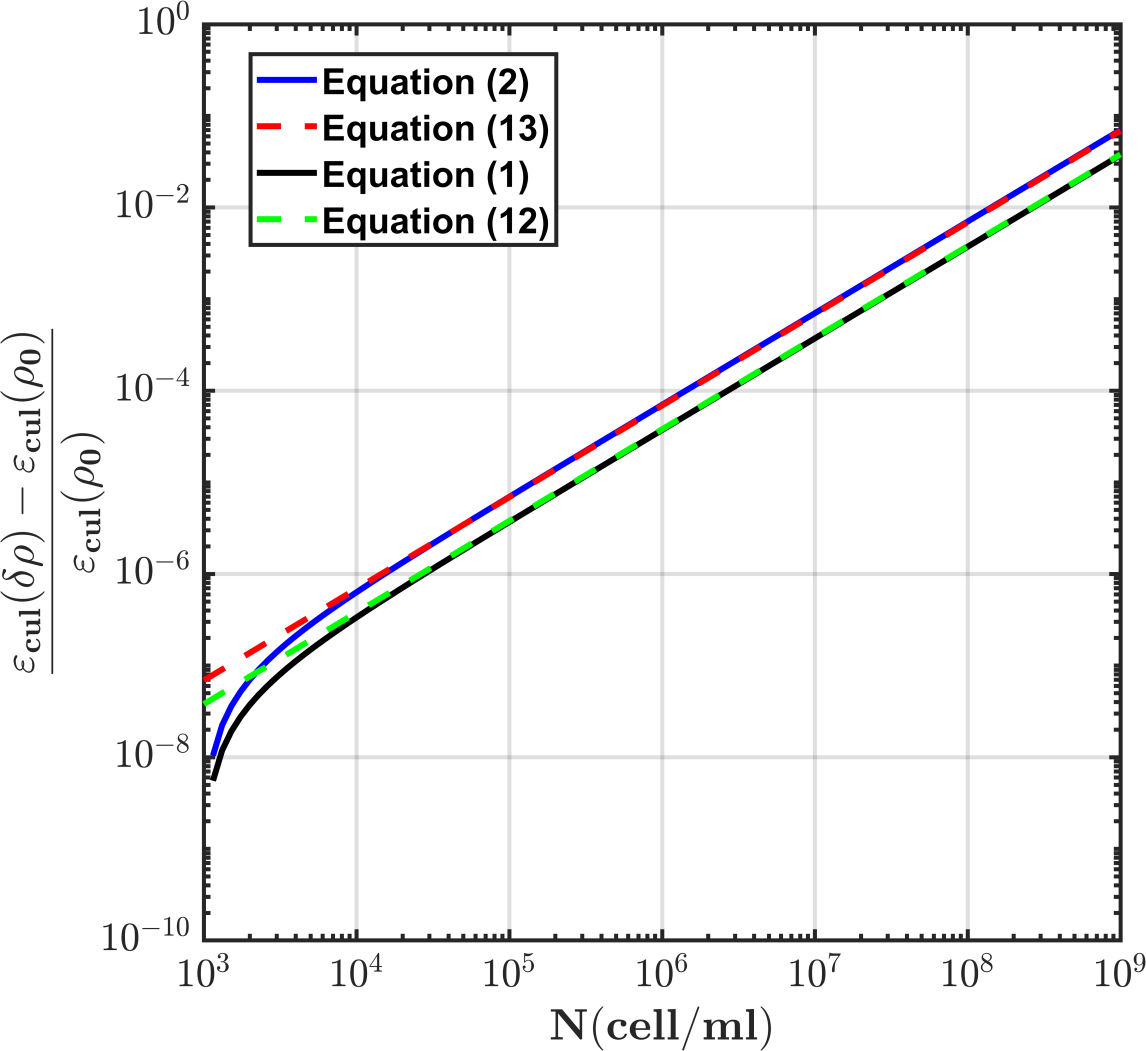
Changes in the dielectric permittivity of the cell culture as a function of the cell concentration for the spherical (Equation (2)) and spheroidal models (Equation (1)) at a frequency of 50 kHz, as well as their corresponding linear fit.

The theoretical model presented in Section 2 allowed us to find the variations in the expected relative dielectric permittivity of the cell culture growth as a function of the cell concentration. From the model and the results shown in Figure 3 and Figure 4 for the sphere and the spheroidal model, the expected evolution of the relative dielectric changes in the culture medium as a function of the cell concentration at a frequency of 50 kHz is shown in Figure 8. The volume fraction ρ of the cell concentration is related to the cell concentration *N* (cells/mL), assuming that ρ=N×V, where *V* is the volume of each cell. From the results in Figure 8 and the linear approximation, the following relationships can be established for the spheroidal and spherical model, respectively:

(8) $$\frac{1}{\epsilon }\frac{\partial \epsilon }{\partial N}\approx 3.8\times 10^{-11};$$

(9) $$\frac{1}{\epsilon }\frac{\partial \epsilon }{\partial N}\approx 6.9\times 10^{-11}$$

and, in consequence, for the spheroid and spherical model, respectively:

(10) $$\frac{V_{d}}{V_{0}}\approx 31.3\times 10^{-11}N;$$

(11) $$\frac{V_{d}}{V_{0}}\approx 56.9\times 10^{-11}N$$

where *N* is the cell concentration, and we considered the calibration constant $k_{M}$ from Equation (A4).

**Figure 9:**
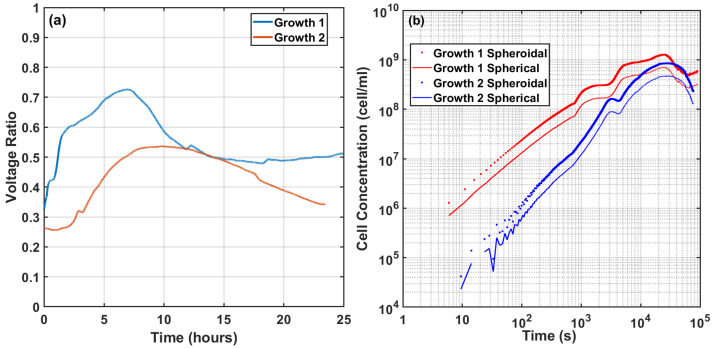
(**a**) Differential voltage ratio measurements over time during two cell growth experiments and (**b**) the derived evolution of cell concentration over time for the spherical and spheroidal models for the two cell growth experiments.

Once the system was calibrated and its sensitivity was determined, a controlled cell culture growth measurement was conducted. A description of the calibration system and the experimental setup is reported in Appendix A.2. To perform this experiment, the two flasks were filled with 45 mL of LB broth from Gibco and placed in an incubator for 3 h so that the whole system warmed up to 37 ${}^{\circ }$C. Once the system was thermally stabilized, the bridge was adjusted to obtain a minimum output voltage, and one of the flasks was inoculated with *E. coli* from ATTC 8739 from WDCM 00012 Vitroids, and culture cell growth was started. The experiment ran for approximately 24 h, and the sampling rate was 7 s. The experiment was performed twice, and Figure 9a shows the measured differential voltage over time. In order to reduce the noise, the output voltage was filtered with a moving average of 10 samples, which roughly corresponded to an integration time of one minute. It was observed that the growth patterns in both experiments followed a similar behavior that was common to cell culture growth. After a flat period, an exponential growth rate followed until reaching saturation.

Figure 9b shows the derived cell culture concentration as a function of time using the sensitivity relationships in Equations (10) and (11) for the two geometrical models of the cells. The final cell concentration was verified through the cell-counting method, and it was of the order of $10^{9}$ cells/mL. The results showed that the spheroidal model provided a better prediction of the cell concentration at saturation, and the sensitivity of the system was of the order of $10^{6}$ cells/mL.

## 5. Discussion

Bioparticle detection is a vital aspect of evaluations of the quality of food and water, in the pharmaceutical industry, and so on. There have been many efforts to describe the dielectric properties of bioparticles as an alternative method for bacteria detection and sensing, as they are simple to use and cost effective. The experimental results show that the theoretical model and, in particular, the spheroidal one provided a good model for predicting the evolution of the changes in the dielectric properties of the growth of a cell culture. The results are significant in the sense that, on one hand, they validate the underlying model for the dielectric constant of a single cell, although the observations were made at the macroscopic level of a cell culture, and on the other hand, they show how, through the well-known measurement method of the capacitive Weathstone bridge, it is possible to measure cell concentration.

We acknowledge that the sensitivity of the setup was limited to measuring cell concentrations above the level of $10^{7}$ cells/mL. Better sensitivity results could be achieved with more sophisticated conditioning electronics that have been developed for the specific field of capacitive sensors and, in particular, capacitive-to-digital conversion [25]. Nevertheless, the achieved sensitivity is better than the initial design objective. In order to have more accurate data on the evolution of the dielectric properties of a cell culture as it grows, future experiments will be focused on combining independent parallel monitoring of cell growth with the capacitive Wheatstone bridge system. This process is not simple, as taking samples out of a culture flask during the growth process interferes with the measurements. A potential solution could be the addition of an OD-600 system, but it has to be carefully designed in order not to affect the capacitive measurement.

## 6. Conclusions

Having a proper model to define the dielectric properties of bioparticles plays a crucial role in their detection and sensing. Two different geometrical models were introduced to estimate the dielectric variation during duplication in an *E. coli* culture. It was shown the dielectric changes in the culture were higher at a low frequency. Therefore, an experimental setup based on a Wheatstone bridge was devised to detect these changes at 50 kHz and to validate the developed method. The system’s setup and calibration procedures were explained thoroughly. Then, cell culture growth was measured based on the dielectric changes in the culture. 

## Figures and Tables

**Figure A1:**
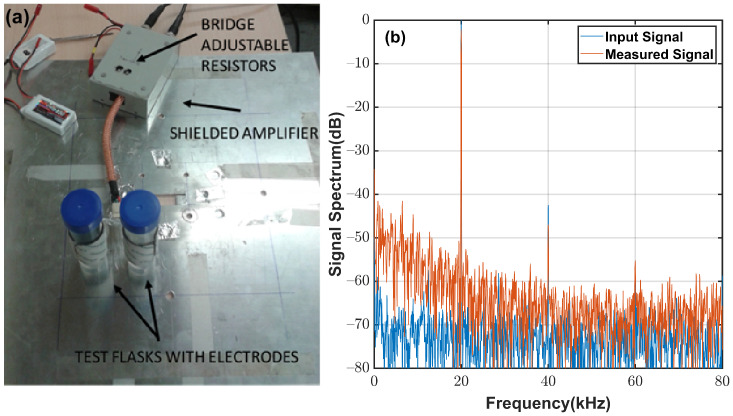
(**a**) Test setup for measuring the noise sensitivity of the system and (**b**) the spectra of the input signal (blue) and measured signal (red).

**Figure A2:**
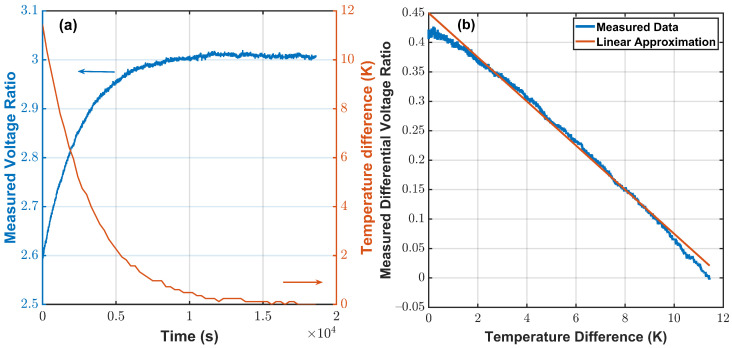
(**a**) Time evolution of the difference in water temperature between the flasks and the measured voltage; (**b**) differential voltage as a function of temperature. The blue line corresponds to the measured data and the red line corresponds to the linear approximation.

**Figure A3:**
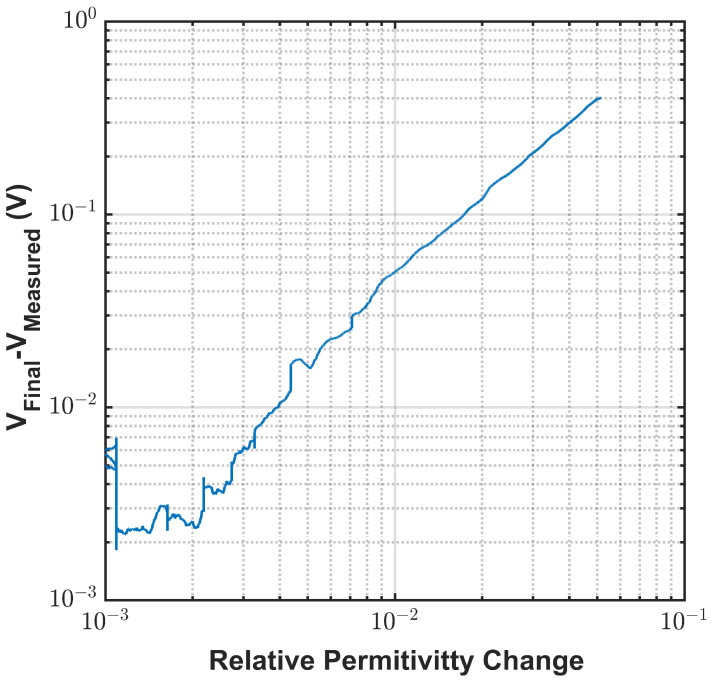
Relative differential voltage measurements as a function of relative changes in permittivity.

**Figure A4:**
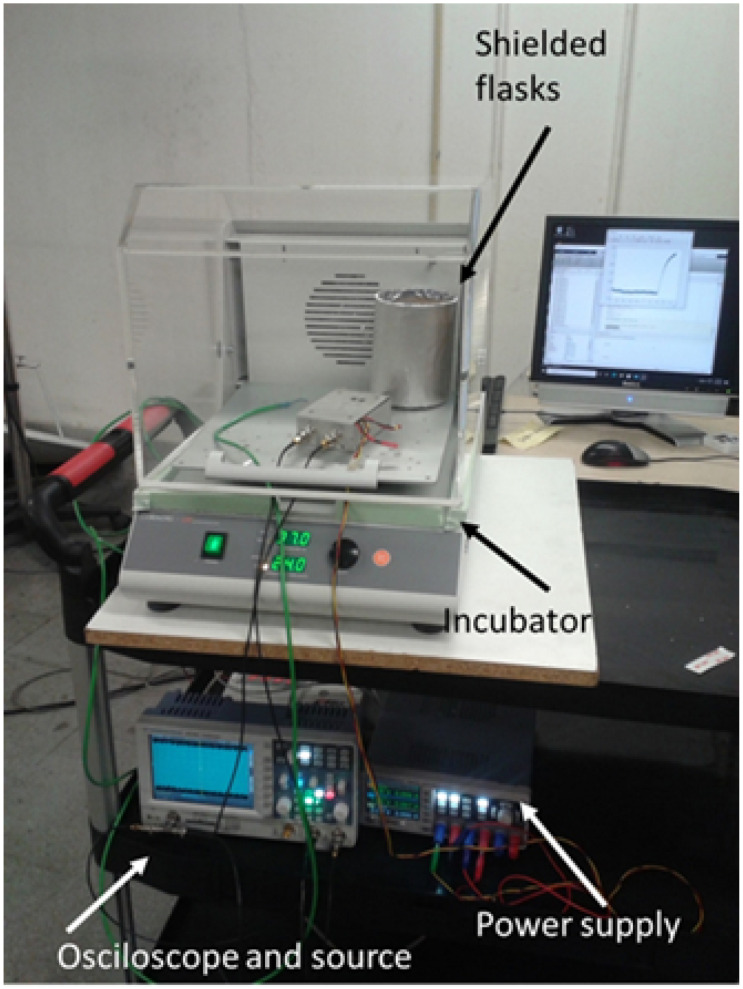
Measurement setup for the monitoring of cell culture growth. Two flasks were shielded with a conducting cylinder to reduce the noise level.

## Data Availability

Not applicable.

## Abbreviations

The following abbreviations are used in this manuscript:

OD	Optical diffusion
CMMR	Common-mode rejection ratio
GCM	Common-mode gain
GD	Differential gain
LB Broth	Lysogeny broth

## Appendix A

### Appendix A.1. System Noise

To evaluate the noise level of the measurement system, the setup in Figure A1a was used. The two flasks were filled with distilled water, and the bridge was balanced using the adjustable resistor until the measured voltage was minimized. Figure A1b shows the normalized spectrum of the input and output signals. The 1/f noise spectrum of the amplifier and a harmonic signal at 100 kHz were considered. The noise level of the output signal was about 60 dB below that of the measured signal, and no noticeable sources of interference were found at the measurement location.

### Appendix A.2. System Calibration

The system’s sensitivity and calibration are described in this section. The following procedure was followed: One of the flasks was filled with distilled water at room temperature, and the other was filled with cold distilled water. The evolution of the temperature of the cold water was monitored by immersing a temperature sensor inside the flask above the electrode level, and a second sensor monitored the room temperature. The variations in the relative dielectric constant of distilled water with temperature are well characterized, and at the frequency of 50 kHz, they have an approximately linear behavior [26]:

(A1) $$\epsilon_{r}\left(T\right)=87.74-0.4008\times T$$

where *T* is the temperature in Celsius, and

(A2) $$\frac{1}{\epsilon_{r}}\frac{\partial }{\partial T}\epsilon_{r}=-4.5\times 10^{-3}$$

Figure A2a shows the evolution of the difference in water temperature between the two flasks, as well as the measured voltage once the bridge was initially balanced by minimizing the output voltage. It took about 5 h to balance the two temperatures, and the exponential decay in the evolution of the temperature difference produced an imbalance in the bridge, which caused an increment in the measured differential voltage. In Figure A2b, the evolution of the differential voltage with the temperature difference is shown together with a linear approximation, where the slope is

(A3) $$\frac{1}{V_{0}}\frac{\partial V_{d}}{\partial T}=-0.0375$$

In this way, the unknown proportionality constant $k_{M}$, which relates the relative changes in the differential voltage measurements and the relative changes in dielectric permittivity, can be determined. This constant encompasses both the bridge sensitivity and the system gain, and it can be found as follows:

(A4) $$k_{M}=\frac{0.00375}{4.54\times 10^{-3}}=8.24$$

Finally, Figure A3 shows the measured differential voltage as a function of the relative changes in the dielectric constant based on Equation (A1). It was observed that sensitivity to changes of $2\times 10^{-3}$ in the relative dielectric constant was achieved, which is within the system’s objective for measuring a cell concentration of $10^{7}$ cells/mL. Notice that, given the amplifier’s differential gain, the value of $k_{M}$ shows that the bridge was not operating under the maximum sensitivity conditions. This was probably due to an increase in the capacitance produced by the cables connecting the flasks to the electronic circuitry, which was not considered when choosing the values of the bridge resistors. Nevertheless, given that the overall system sensitivity met the design goal, the system was not modified.

Figure A4 shows the measurement setup for the monitoring of cell culture growth. A CORNING-LSE incubator was used to accurately control the temperature, and it provided shaking oscillations that prevented growing cells from being deposited at the bottom of the flask while providing recirculation of oxygen and nutrients. The electronics with the differential amplifier were placed inside the incubator; in this way, it is also thermally stabilized. An increase in the electromagnetic noise was observed, especially when the incubator shaker was turned on. To reduce the noise levels to the acceptable values shown in Figure A1b, it was sufficient to shield the two flasks with a conducting cylinder.
